# Climatic fluctuations and malaria transmission dynamics, prior to elimination, in Guna Yala, República de Panamá

**DOI:** 10.1186/s12936-018-2235-3

**Published:** 2018-02-20

**Authors:** Lisbeth Amarilis Hurtado, José E. Calzada, Chystrie A. Rigg, Milagros Castillo, Luis Fernando Chaves

**Affiliations:** 1 Unidad de Análisis Epidemiológico y Bioestadísticas, Instituto Commemorativo Gorgas de Estudios de la Salud, Panamá, República de Panamá; 2 Departamento de Investigación en Parasitología, Instituto Commemorativo Gorgas de Estudios de la Salud, Panamá, República de Panamá; 3 Instituto Costarricense de Investigación y Enseñanza en Nutrición y Salud (INCIENSA), Apartado 4-2250, Tres Ríos, Cartago Costa Rica; 4 Programa de Investigación en Enfermedades Tropicales (PIET), Escuela de Medicina Veterinaria, Universidad Nacional, Apartado Postal 304-3000 Heredia, Costa Rica

**Keywords:** NDVI, Malaria elimination, *Gunas*, *Plasmodium vivax*, Wavelets, Seasonal autoregressive, Climate change, El Niño Southern Oscillation, Poverty, *Anopheles albimanus*

## Abstract

**Background:**

Malaria has historically been entrenched in indigenous populations of the *República de Panamá*. This scenario occurs despite the fact that successful methods for malaria elimination were developed during the creation of the Panamá Canal. Today, most malaria cases in the *República de Panamá* affect the *Gunas*, an indigenous group, which mainly live in autonomous regions of eastern Panamá. Over recent decades several malaria outbreaks have affected the Gunas, and one hypothesis is that such outbreaks could have been exacerbated by climate change, especially by anomalous weather patterns driven by the EL Niño Southern Oscillation (ENSO).

**Results:**

Monthly malaria cases in Guna Yala (1998–2016) were autocorrelated up to 2 months of lag, likely reflecting parasite transmission cycles between humans and mosquitoes, and cyclically for periods of 4 months that might reflect relapses of *Plasmodium vivax*, the dominant malaria parasite transmitted in Panamá. Moreover, malaria case number was positively associated (P < 0.05) with rainfall (7 months of lag), and negatively with the El Niño 4 index (15 months of lag) and the Normalized Difference Vegetation Index, NDVI (8 months of lag), the sign and magnitude of these associations likely related to the impacts of weather patterns and vegetation on the ecology of *Anopheles albimanus*, the main malaria vector in Guna Yala. Interannual cycles, of approximately 4-year periods, in monthly malaria case numbers were associated with the El Niño 4 index, a climatic index associated with weather and vegetation dynamics in Guna Yala at seasonal and interannual time scales.

**Conclusion:**

The results showed that ENSO, rainfall and NDVI were associated with the number of malaria cases in Guna Yala during the study period. These results highlight the vulnerability of *Guna* populations to malaria, an infection sensitive to climate change, and call for further studies about weather impacts on malaria vector ecology, as well as the association of malaria vectors with *Gunas* paying attention to their socio-economic conditions of poverty and cultural differences as an ethnic minority.

**Electronic supplementary material:**

The online version of this article (10.1186/s12936-018-2235-3) contains supplementary material, which is available to authorized users.

## Background

Malaria was one among many infectious vector-borne diseases that were studied and then locally eliminated as a necessary step for the building of the Panamá Canal [1, 2], and a disease kept under control during the development of the Canal Zone as a US colonial possession [3–5]. Pioneering research on infectious diseases in the Canal Zone showed that malaria was a major problem among indigenous populations [6, 7], a situation that, unfortunately, is currently maintained [8]. A major factor shaping this situation is the poor housing quality of the *Gunas* [9] and their different cultural practices [10], where a major point of pride has been keeping to a minimum the influence of Hispanic culture on *Guna* traditions [11]. In fact, after the independence of Panamá from Colombia a series of concessions by the Panamanian government to exploit natural resources on the ancestral lands of the *Gunas*, in a fashion that excluded the *Gunas* from their economic benefits, coupled with a series of actions that forcibly tried to incorporate the *Gunas* into mainstream Panamanian culture, led to a popular uprising and armed revolt in 1925: *La Revolución Guna* led by Nele Kantule, a major leader in the *Guna* struggle for self-determination and right to keep their ancestral heritage. Resolution of this conflict was negotiated through a peace treaty whereby the Panamanian government committed itself to grant special rights to the native communities of Panamá, rights finally granted with the creation of the autonomous indigenous region of Guna Yala, originally named San Blas, in 1938 [12].

The autonomy granted to the *Gunas*, nevertheless, has not empowered this ethnic group to improve its socio-economic and health indicators. Indeed, although Panamá is one of the countries with a high likelihood of eliminating malaria in the near future [13, 14], malaria is by far the most important infectious vector-borne disease affecting the *Gunas*, and this probably reflects the vulnerability of this ethnic group to malaria transmission as a socio-economically marginalized population [8, 15]. Today, around 90% of the malaria cases in Panamá come from the *Gunas*, despite being less than 3% of the total population in Panamá, and near 40% of the cases occur in the Comarca Guna Yala [16]. In this area the dominant malaria vector is *Anopheles albimanus* [16, 17], while the dominant parasite is *Plasmodium vivax*, which consistently accounts for over 90% of the cases [18, 19]. In this scenario, where malaria transmission has been declining over recent years [8], a major concern is the role that climate change might have on malaria transmission [20, 21], given the high vulnerability of the *Gunas* as a socially marginalized ethnic group. For example, with climate change-induced sea level rise it is expected that the core of the *Guna* population, who inhabit small islands in the Caribbean Sea, will need to be relocated to the mainland of Guna Yala [22], mainly to Cartí, an area with very active malaria transmission, unlike the islands where transmission is rare [16]. A previous experience of relocating *Gunas* inside Panamá lacked an environmental health impact assessment, and soon after *Gunas* from the Comarca Madugandi were relocated to the shores of Lake Bayano, several vector-borne infections affected this relocated *Guna* population [23]. Nowadays, the *Gunas* have the largest malaria burden in Panamá [8]. In that sense, it is important to understand different factors affecting malaria transmission in Guna Yala. Previous research has shown that in the nearby autonomous region of Madungandi, also inhabited by the *Gunas*, extreme conditions in the El Niño Southern Oscillation (ENSO) were associated with an exacerbation of malaria transmission in this region [8]. This research tests the hypothesis that ENSO and weather fluctuations might be associated with changes in malaria transmission in the Comarca Guna Yala. This research uses tools for time series analysis to assess the impact of ENSO, meteorological fluctuations (rainfall and temperature) and vegetation dynamics on malaria transmission dynamics.

## Methods

### Study site

**Fig. 1:**
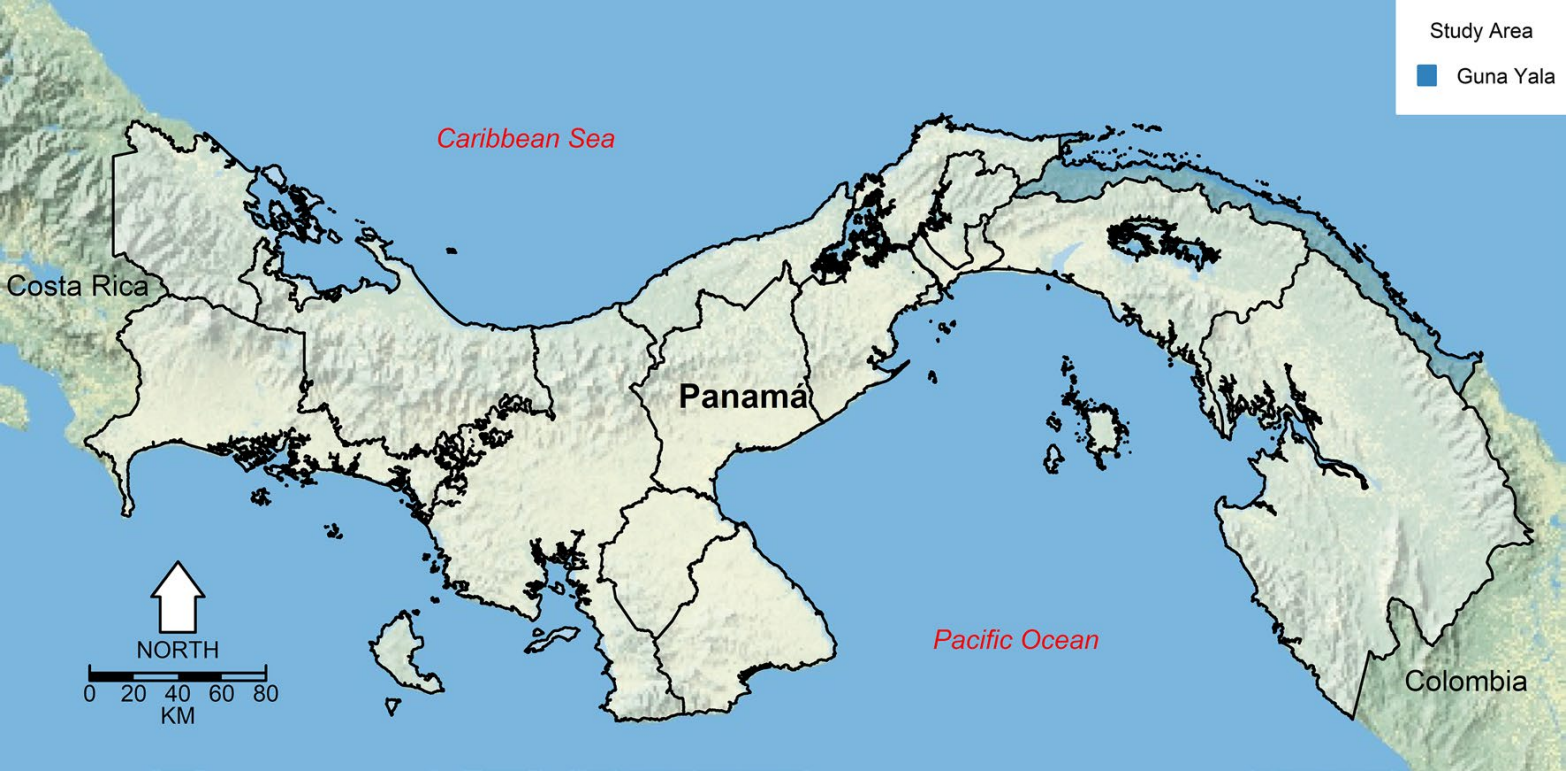
Map of the República de Panamá, highlighting the location of Comarca Guna Yala. This map was made using as base a public domain map from the US National Park Service [84]

The Comarca Guna Yala is located in northeastern Panamá, facing the Caribbean Sea, and bordering Colombia in the southeast (Fig. 1). The climate is classified as sub-equatorial with a dry season [24]. Like the rest of Panamá there is little seasonal variability in temperature which oscillates between 26 and 27 °C, with an unimodal rainfall seasonal pattern with a dry (December to April) and long rainy season (May to November). The total population of Guna Yala is around 37,000 people, with 19,500 females and 17,500 males, around 50% of the population are under 18 years of age, and around one-fifth of the population are children under 5 years old. Poverty is a major problem in the region, for example, 91.4% of the people are poor, one of the highest in Panamá according to a multidimensional poverty index developed by Panamá’s Ministry of Finance [25], and closely related to the fact that nearly 80% of the population is dedicated to subsistence farming and fishing [26]. More specifically, there are large differences in quality of life parameters, where the *Gunas* have a life expectancy of 71 years well below the average for the population of Panamá (78 years), and, in general most socio-economic indicators lag well behind those of the non-indigenous population [26].

### Climatic and landscape covariates

Several weather stations are located in Comarca Guna Yala, but only two have long-term rainfall records (Additional file 1: Figure S1). Therefore, to assess the impact of local weather fluctuations, data from globally interpolated gridded datasets were used. For temperature and rainfall, respectively, data from GHCN/CAMS 2 m and CMORPH Version 1.0 available at [27] were used. Both of these datasets are available in grids of 0.5°. Data were downloaded for the gridded box bounded between 8.5°N and 9.5°N and 79.25°W and 77.75°W. To quantify the impacts of global climatic fluctuations the El Niño 4 time series was employed, an index for ENSO, a global climatic phenomena associated with extreme weather in Panamá [8]. Niño 4 data were downloaded from the US National Oceanic and Atmospheric Administration (NOAA) Climate Prediction Center [28]. The NOAA data were collected from the area delimited by 5°N–5°S and 160°E–150°W of the Pacific Ocean. All these time series were available from January 1998 to December 2016.

As a proxy of vegetation data, information from the MODIS land products database was extracted. Images for the Normalized Difference Vegetation Index (NDVI) from the monthly 1-km resolution vegetation (M*D13A3) product, courtesy of the NASA Land Processes Distributed Active Archive Center (LP DAAC), USGS/Earth Resources Observation and Science (EROS) Center, Sioux Falls, South Dakota [29], were employed. To download the images the package MODIStsp for the software R [30] was employed. Further GIS procedures for the downloaded images were made using the package raster also in the statistical software R, where each monthly image was clipped using a shapefile for Comarca Guna Yala, then stacked into a geotiff, from which the average and standard deviation for each clipped polygon was computed, thus generating a time series. MODIS NDVI based products were only available from January 2000 to December 2016.

### Malaria data

Monthly malaria cases for Comarca Guna Yala, from January 1998 to December 2016, were obtained from the *Departamento de Control de Vectores*, *Ministerio de Salud*, *República de Panamá*. The time series records malaria cases from all over Comarca Guna Yala, although epidemic foci occurred in selected locations [16]. The time series only considers confirmed malaria cases by the examination of Giemsa-stained blood smears prepared by the thick smear method [6]. Routinely, all positive slides, and 10% of the negative slides, are confirmed by the Public Health Central Reference Laboratory of the Gorgas Laboratory [8]. This microscopic diagnostic has shown a consistent sensitivity and specificity, close to 100% in each case, confirming the quality of the data. With the exception of the 2002–2006 outbreak, where 12% of the cases were due to *Plasmodium falciparum* [18], over 95% of the cases are consistently due to *P. vivax*. For the subsequent time series analysis the raw number of malaria cases were analysed, provided this time series did not have trends that required the estimation of the malaria incidence rate for de-trending [31].

### Statistical analysis

#### Malaria cases, weather and vegetation seasonality

Seasonality in malaria cases and the covariate time series was studied using box diagrams for each month [32].

#### Malaria cases time series correlation structure and association with climatic variables

For the analysis, a protocol previously applied to study malaria in Panamá was followed [8]. Briefly, the protocol starts by inspecting the autocorrelation function (ACF), i.e., the time series correlation with itself through time, and the partial autocorrelation function (PACF), i.e., the correlation between consecutive time lags [31]. With information about the significant time lags, a null autoregressive model with no covariates, was built. This null model was used to pre-whiten the time series from all covariates using the Kalman filter [31]. Pre-whitening is a process where any common autoregressive structure is removed from ancillary time series in order to study its patterns of association with a focal time series [31]. Residuals from the autonomous null model and pre-whitened residuals from covariates were used to estimate cross-correlation functions (CCFs) of malaria incidence with each one of the covariates. The CCFs indicate lags at which malaria and the covariates were correlated, which were subsequently used to build models with covariates. The model that included all significant covariates was simplified by a process of backward elimination guided by the minimization of the akaike information criterion (AIC), a function that trades-off model goodness-of-fit and parameter number, and whose minimization can be used to select among models with a similar number of parameters [31]. For the best model error assumptions were verified using standard procedures for time series analysis [31].

#### Non-stationary patterns of association in the time–frequency domain

The association of cycles in time series over time can be studied using cross wavelet coherence analysis [33, 34]. Here, wavelet-based analysis was used to determine at a particular frequency and time in Niño 4 index and malaria, and also to see if these time series were associated with climatic covariates, i.e., temperature and rainfall, and the mean and standard deviation (SD) of NDVI in Comarca Guna Yala.

## Results

**Fig. 2:**
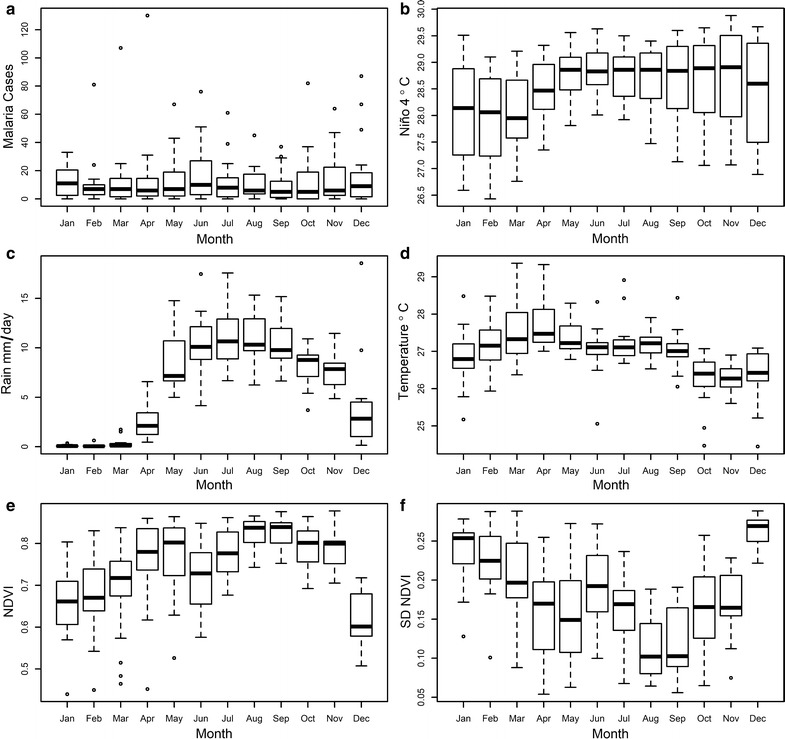
Seasonal patterns. **a** Malaria (**b**) Niño 4 (**c**) rainfall (**d**) temperature (**e**) average NDVI (**f**) SD NDVI. In the boxplots, middle bars indicate median values

During the study period (1998–2016) there was a total of 3082 malaria cases in Guna Yala, with a monthly average (± SD) of 13.52 ± 14.03. Seasonal patterns in the studied time series are presented in Fig. 2. For malaria cases (Fig. 2a), there was no clear seasonality, with the number of cases being low, around 10 cases per month, throughout the year. In all months but January there were epidemics of 30 or more cases (Fig. 2a). The median Niño 4 index (Fig. 2b) tended to be lower during the first 4 months of the year, January to April, but there was relatively little seasonal variability in this index. Meanwhile, rainfall (Fig. 2c) had a clearly marked seasonality, where December to April are dry, and the rest of the year is rainy, reaching a peak in July. Temperature (Fig. 2d) has its seasonality contrasting with rainfall, where the dryer months are the hottest months, temperatures reaching a monotonic peak in April. The NDVI showed a bimodal pattern (Fig. 2e) reaching a first peak in May and then in September, with an absolute minimum in December. By contrast, seasonality in the NDVI SD reached peaks when the NDVI is lowest, e.g., in January and December (Fig. 2f).

**Fig. 3:**
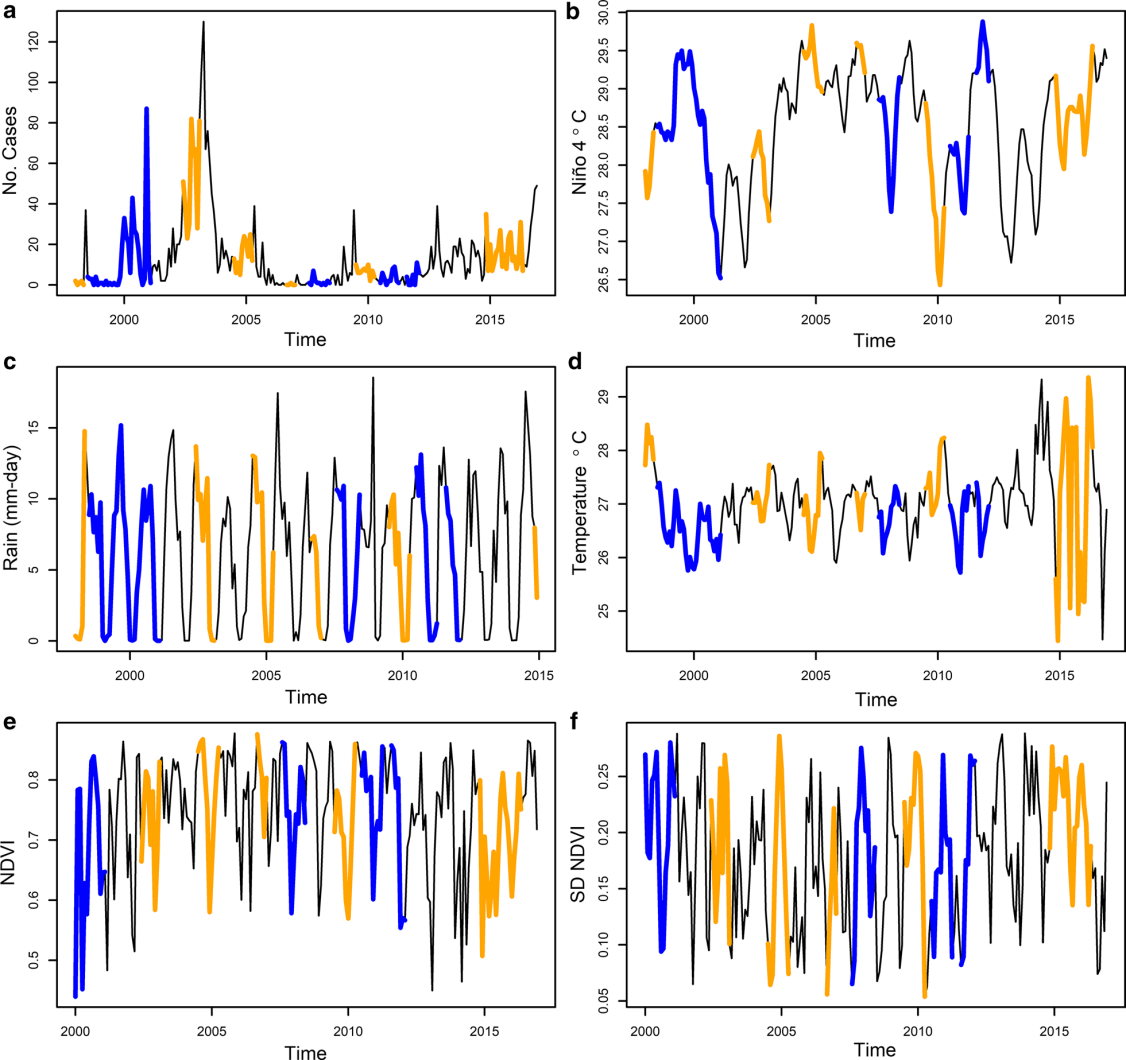
Monthly time series. **a** Malaria (**b**) Niño 4 (**c**) rainfall (**d**) temperature (**e**) average NDVI (**f**) SD NDVI. In all panels, blue indicates the cold phase of the El Niño southern oscillation, while orange the hot phase

Figure 3 shows the studied monthly time series highlighting ENSO phases. At the beginning of the study period (2000–2004), two malaria epidemics occurred during the cold and hot phases of ENSO, and overall it seems from the plot that malaria cases increased with ENSO, especially during the hot phase (Fig. 3a). During these periods the Niño 4 index (Fig. 3b) has its maximum and minimum, while rainfall tends to decrease during the hot phase of ENSO (Fig. 3c) a time when temperature increases in Guna Yala (Fig. 3d). For NDVI there is no clear increase or decrease, both on its mean value (Fig. 3e) or SD (Fig. 3f) that could be associated with specific ENSO phases.

**Fig. 4:**
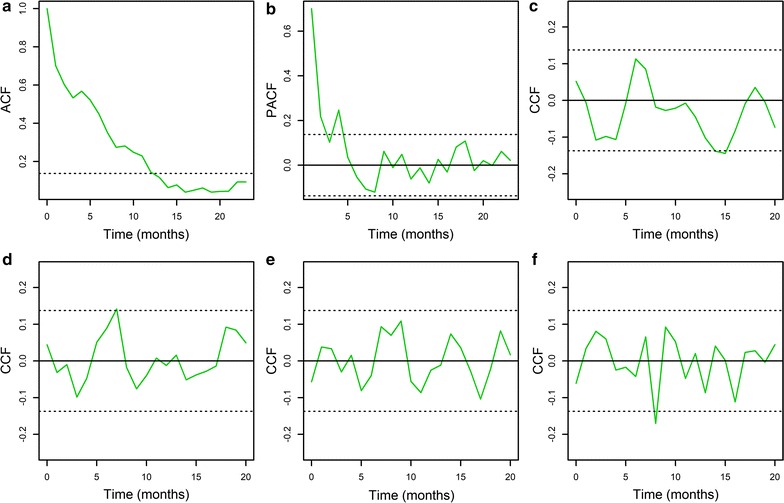
Correlation functions for the 2000–2016 monthly time series. **a** Malaria time series autocorrelation function (ACF). **b** Malaria time series partial autocorrelation function (PACF). Cross correlation function (CCF) between malaria and **c** Niño 4 (**d**) rainfall (**e**) temperature (**f**) average NDVI. In the plot panels, orange lines indicate the value of the correlation function, the black solid line indicates a correlation value of 0, while the dotted lines indicate 95% confidence intervals within which correlations are expected by chance. Time lags in the x axis of all panels are in months

Autocorrelation patterns in the malaria time series, and its association with the different covariates are presented in Fig. 4, considering data between January 2000 and December 2016. Only for this period there was information about all covariates, while there was data for Niño 4, rain and temperature for 1998–2016, and for comparison, results for this time period are presented in Additional file 2: Figure S2. Figure 4a shows that malaria had a descending autoregressive pattern, suggestive of a significant degree of autocorrelation in the time series. A similar pattern is also observed for the time series between 1998 and 2016 (Additional file 2: Figure S2A). More specifically, the time series had a significant partial autocorrelation (Fig. 4b) at times lags 1, 2 and 4 months, which were also significant when considering data from 1998 and 1999 (Additional file 2: Figure S2B). Malaria cases were negatively associated at a time lag of 15 months (Fig. 4c, Additional file 2: Figure S2C), and positively with rain at 7 months of lag (Fig. 4d, with no significance when considering data for 1998–2016, see Additional file 2: Figure S2D), and no significant association was observed with temperature independently of the studied time frame (Fig. 4e, Additional file 2: Figure S2E). Malaria was negatively associated with NDVI at 8 months of lag (Fig. 4f) and no association was observed with the SD of NDVI (Additional file 3: Figure S3). The information from the correlation patterns was used to build seasonal autoregressive models (Table 1), which considered 2 monthly time lags as autoregressive, and a seasonal autoregressive component with 4 months of lag (Fig. 4b). One model did not include any covariate, while another included the 3 covariates that were identified as significantly associated with the number of malaria cases, i.e., the El Niño 4 index with 15 months of lag (Fig. 4c), rainfall with 7 months of lag (Fig. 4d) and NDVI with 8 months of lag (Fig. 4f). This model with all covariates had the minimum AIC when compared with the model without covariates, and simpler models (Table 1).

**Table 1:** Time series model selection

Parameters (lag)	AIC
Intercept, AR(1), AR(2), SAR(4)	1571.27
Intercept, AR(1), AR(2), SAR(4), Niño 4(15), rain(7), NDVI(8)	*1559.89*
Intercept, AR(1), AR(2), SAR(4), Niño 4(15), rain(7),	1568.70
Intercept, AR(1), AR(2), SAR(4), Niño 4(15), NDVI(8)	1562.46
Intercept, AR(1), AR(2), SAR(4), rain(7), NDVI(8)	1562.75
Intercept, AR(1), AR(2), SAR(4), Niño 4(15)	1568.41
Intercept, AR(1), AR(2), SAR(4), rain(7)	1569.72
Intercept, AR(1), AR(2), SAR(4), NDVI(8)	1568.04

Parameters indicate the parameters considered in each model

*AIC* Akaike information criterion is minimized for the best model, indicated in italic type. Parameters include: *AR* autoregressive, *SAR* seasonal AR, Niño 4, rain and NDVI, lags are in months

Parameter estimates for the best model are presented in Table 2. All coefficients were statistically significant (P < 0.05). Meanwhile, as observed in the CCFs, the association of monthly malaria case number was negative with the El Niño 4 index (Fig. 4c), NDVI (Fig. 4f) and positive with rainfall (Fig. 4d), the relationship of monthly malaria case number being largest with NDVI (Table 2).

**Table 2:** Parameter estimates for the best time series model explaining the number of malaria cases in Comarca Guna Yala (2000–2016), Panamá

Parameter (Lag)	Estimate	SE	Z
Intercept	37.896	7.854	4.825*
AR(1)	0.532	0.072	7.389*
AR(2)	0.16	0.074	2.162*
SAR(4)	0.263	0.088	2.989*
Niño 4(15)	− 5.61	2.503	− 2.241*
Rain(7)	0.508	0.237	2.143*
NDVI(8)	− 30.946	9.367	− 3.304*
Error variance	153.5		

Parameters include: *AR* autoregressive, *SAR* seasonal AR, Niño 4, rain and NDVI, lags are in months

* Statistically significant, P < 0.05

**Fig. 5:**
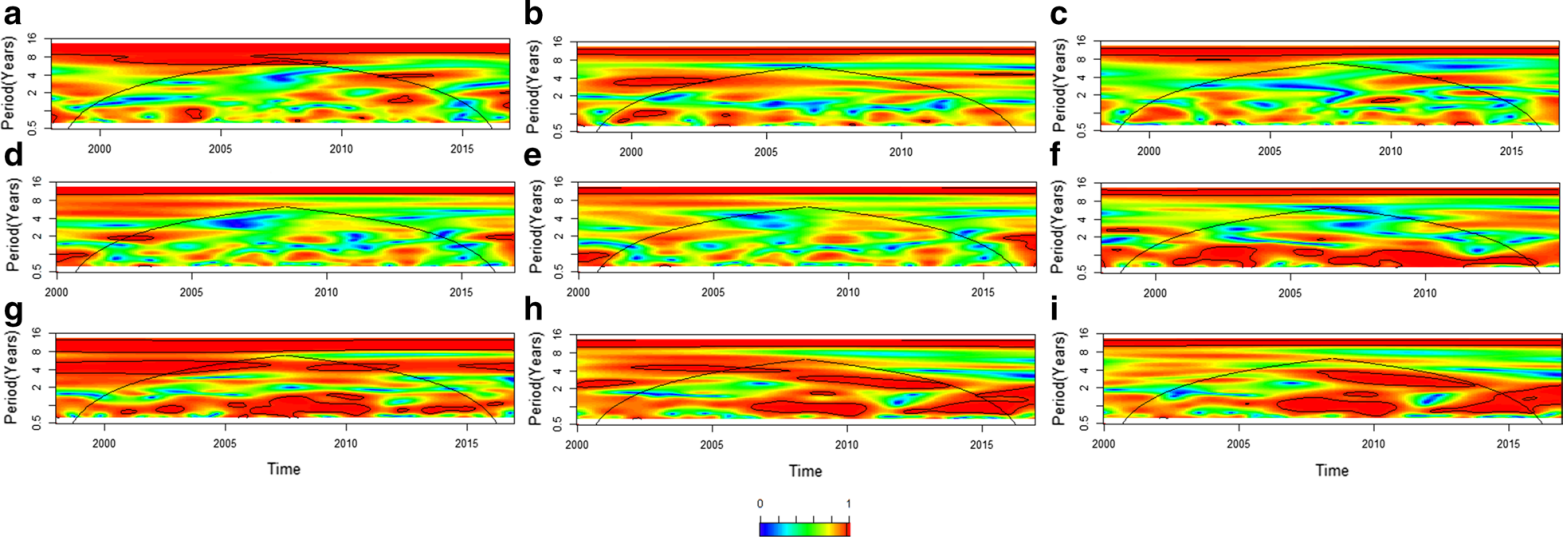
Cross wavelet coherence analysis for monthly time series. Panels show the cross-wavelet coherence between: **a** malaria and the El Niño 4 index, NI (**b**) malaria and rainfall (**c**) malaria and temperature (**d**) malaria and NDVI (**e**) malaria and SD of NDVI (**f**) NI and rainfall (**g**) NI and temperature (**h**) NI and NDVI (**i**) NI and SD of NDVI. A coherence scale is presented on the right-hand side of the figure, which goes from zero (blue) to one (red). Red regions in the plots indicate frequencies and times for which the two series share power (i.e., variability). The cone of influence (within which results are not influenced by the edges of the data) and the significant coherent time–frequency regions (P < 0.05) are indicated by black solid lines. Note that cross-wavelet analysis including NDVI data are for 2000–2016, while all other analyses are for 1998–2016

As suggested by the changing significance of the association between malaria cases and, for example, rainfall (Fig. 4d and Additional file 2: Figure S2), the association between malaria case number and the covariates were considered highly non-stationary, changing on time and frequency (Fig. 5). This result is confirmed when looking at the wavelet cross-coherence between the monthly number of malaria cases and the El Niño 4 index (Fig. 5a), which suggests interannual cycles, of 4-year periods or more, between the two time series are associated. The association of malaria with the other time series was more localized, and prominent at seasonal time scales (1-year period) or biennial, as was the case with rainfall (Fig. 5b), and to a lesser extent with temperature (Fig. 5c), NDVI (Fig. 5d) and the SD of NDVI (Fig. 5e). Meanwhile, the cross wavelet coherence analyses suggest that impacts of El Niño 4 impacts of the climatic and vegetation covariates at Guna Yala were widely significant (P < 0.05) during the study period. For rainfall, associations were stronger at the seasonal and biennial time scales (Fig. 5f), while for temperature a similar pattern was observed, but also with a significant coherence at interannual time scales of 4 years or more (Fig. 5g). Finally, NDVI (Fig. 5h) and SD of NDVI (Fig. 5i) were associated seasonally and interannually, for cycles of 4-year periods, with the El Niño 4 index.

## Discussion

Mesoamerica, the geographic region traversing southern México to Panamá, is a region where around 3% of malaria cases occur in the New World [35]. Most countries in Mesoamerica achieved the World Health Assembly (WHA) target for the Millennium development goals (MDG) of reducing by 75% malaria cases when compared with 2000 [35, 36]. Nevertheless, Panamá, despite having the highest economic growth in Mesoamerica [37], was the only country unable to achieve the MDG for malaria reduction. In fact, the 874 malaria cases reported in 2014 represented only a 15.6% decrease in annual malaria cases when compared with 2000 [36]. Most malaria cases, over 85% of the total, occur in indigenous groups that inhabit the autonomous regions of the country, which are socially marginalized and vulnerable populations, and where the use of conventional tools for malaria control is less effective than among other groups [8]. This failure might reflect the alarming social and health inequalities affecting the *Gunas* [26], but also a lack of intercultural understanding [10]; other factors, including geographical isolation, internal movement of *Gunas* across the region and cross-border movement of people between Colombia and Guna Yala, have further constrained the success of efforts to control malaria in this region, thus favouring malaria transmission in an epidemic-prone fashion [16, 19].

Moreover, Guna Yala is highly vulnerable to climate change, mainly because of its geography characterized by an extended coastline on the Caribbean Sea with many inhabited small islands [38]. In fact, global warming-associated rise in sea level and subsequent reduction in surface area of some islands has already caused the displacement of some *Guna* populations to mainland areas [22, 39], where there is a higher risk for malaria transmission [16]. Nevertheless, climate change also affects malaria transmission by impacting the population dynamics of vectors [40] and the relationship of vectors and parasites [41, 42], whose understanding can help to optimize the targeting of control strategies by the Panamanian National Malaria Control Programme. In this sense, this study explored the impact of ENSO, meteorological fluctuations (rainfall and temperature) and vegetation on malaria transmission dynamics in Guna Yala.

During the study period (1998–2016), the monthly number of malaria cases was relatively low and homogenously distributed throughout the years. Epidemics were observed in all months, except for January, a behaviour that can be exploited to intensify control activities or implement intervention measures such as mass drug administration during that month, as done elsewhere [43]. The monthly malaria cases autocorrelation observed at 1 and 2 months lag likely reflect parasite transmission cycles between humans and *Anopheles* spp. mosquitoes [44, 45]. Similarly, the 4 months lag is likely related to relapses of *P. vivax* [46–49], which have been described as occurring between 3 and 7 months with an average of 5 months in *P. vivax* strains from Panamá [50], the dominant malaria parasite transmitted in Panamá, including Guna Yala [16].

National drug policy on malaria in Panamá recommends chloroquine in combination with primaquine as first-line treatment for *P. vivax* infections [18]. The World Health Organization recommends a 14-day course of primaquine to eradicate the liver stage of the parasite and prevent relapse of the disease [18]. However, in most of Panamá, particularly in hard-to-reach areas as Guna Yala, primaquine is administered for only 7 days [51]. Shorter regimens compared to the standard 14-day primaquine have been associated with higher relapse rates [52]. Moreover, due to their traditional beliefs and practices, *Gunas* do not adhere well to treatments they consider foreign to their culture [10]. Thus, many *Guna* patients fail to complete the full dosage, resulting in inadequate blood drug concentration that favours relapse [51].

Environmental covariates with significant associations (P < 0.05) were rainfall, positively associated with 7 months of lag, and cases were negatively associated with the El Niño 4 index (15 months of lag) and the NDVI (8 months of lag). These relatively long delays might emerge as consequence amplifying cycles of transmission, a phenomenon previously described in East Africa [53], where a triggering environmental disturbance has an effect that exacerbates through time via the amplification of those environmental signals, for example, by mosquito vectors [40]. These long lags might also reflect an association with a harmonic of a cycle from a shorter period [54]. In plain words, if there is a cycle of 2 months associated with the life cycle of the parasite, then the incidence can also be expected to be associated at 4, 6 and 8 months, which are harmonics of 2 months. Then if rainfall itself has an autocorrelated pattern, where 7 months is a natural cycle, one can expect the association to be stronger due to a phenomenon known in physics as ‘resonance’ [54, 55] that emerges in coupled oscillators (which metaphorically can be used to describe vector-borne disease, mosquito and rainfall association [56]) and which increases the association at time lags that correspond to rainfall cycles or rainfall impact on malaria transmission. Moreover, here it is important to highlight the extensively validated methods employed for this analysis [31], warrant that these lags are not spurious results. The ENSO is considered a potential driver of malaria transmission in endemic regions across the globe [44, 53, 57–64], and for other tropical diseases in the region [34, 65–67]. Panamá, with extended coastlines in the Pacific and the Caribbean, is highly vulnerable to the impact of this global climatic phenomenon [68]. In fact, ENSO events may trigger heavy rainfall in the Caribbean coast and at the same time severe and prolonged drought in the Pacific Coast [68, 69]. In this sense, this study also found that interannual cycles, of approximately 4-year periods, in monthly malaria case numbers were significantly associated with ENSO, measured by El Niño 4 index. This result is similar to that previously found in the Madungandi autonomous region, a nearby mainland region also inhabited by the *Gunas* [8].

About the other environmental covariates associated with malaria cases in Guna Yala, it is well known that rainfall is necessary to form water pools that are used by *Anopheles* spp. as breeding and larval sites [2, 20, 21]. This is important in Guna Yala as many of the inhabited islands lack fresh water and many temporal water pools suitable for *Anopheles* spp. breeding are formed soon after the rainy season ends [16]. Although temperature has a critical impact on mosquito and parasite traits that determine the transmission potential of malaria [41, 42, 70], this analysis did not find a significant association with malaria cases in Guna Yala. This is probably related to the lack of a marked seasonality that could disrupt *Plasmodium* spp. development in *Anopheles* spp. mosquitoes, with the average temperature of 26.99 °C (range 24.45–29.36 °C] close to 26.00 °C (range 17.00–33.00 °C) a temperature deemed optimal for malaria transmission [42]. Meanwhile, the negative impact of NDVI might be related with local ecological conditions [58, 71], where the excessive increase of vegetation biomass is detrimental for population dynamics of *An. albimanus* and other dominant vector species present in Guna Yala, mainly *Anopheles aquasalis* and *Anopheles punctimacula* [72–74]. Indeed, it is very important to highlight the importance of local ecological conditions for different dominant malaria vector species. For example, *An. albimanus* which is considered the main malaria vector in Guna Yala [16], breeds in a wide variety of aquatic habitats with several types of aquatic vegetation [2, 74], but with a biology sensitive to shade [2] which might explain the negative impact of increased terrestrial vegetation. By contrast, *An. punctimacula* shows preference for shallow waters shaded by coconut palms [16, 17], while *An. aquasalis* breeding sites are mainly mangroves and coastal wetlands, and its abundance has been associated with salinity [75, 76]; ecological characteristics predominant in most *Guna* settlements in Guna Yala. Interestingly, this latter vector species has a restricted distribution in Panamá, but is particularly prevalent in Guna Yala, being the primary vector in some communities of this region [17]. Probably these other vectors, better suited to local habitats observed in Guna Yala, may be responsible for malaria transmission, but that is open to further research.

Finally, some limitations of this study need to be highlighted: for the analysis *P. vivax* and *P. falciparum* data were together as total malaria cases because, with the exception of the 2004 epidemic [18], over 95% of the cases were due to *P. vivax* during the study period. Besides this, there might be some problems in the accuracy of case detection given the passive nature of the malaria surveillance system, a problem common in other malaria-endemic regions [46, 77]. Similarly, the lack of association with temperature might be an artifact of the geographical scale of the study, given that at small geographical scales temperature is important for mosquito biology and the parasite vector interaction [41, 75]. There is the pervasive problem of ignoring other contextual drivers of malaria transmission such as poverty [8, 78, 79], parasite invasion through unplanned migration [19, 80], and the understudied socio-cultural barriers to accept and implement malaria control measures among the *Gunas* [10].

## Conclusions

Results from this study showed that ENSO, rainfall and NDVI were associated with the number of malaria cases in Guna Yala during the study period (1998–2016). These results highlight the vulnerability of *Guna* population to malaria, an infection sensitive to climate change, and call for further studies about weather impact on malaria vector ecology, especially the temporal impact of weather fluctuations on population dynamics [81, 82], something that has not been done for *An. albimanus* in the Comarca Guna Yala of Panamá, as well as the association of malaria vectors with the *Gunas*, paying special attention to their socio-economic conditions of poverty and cultural differences as an ethnic minority. Other biological factors, such as the influx of malaria parasites by the migration of the *Gunas* across their ancestral territory, and other ethnic migrant groups: all of these conditions favour malaria transmission in the landscape inhabited by the *Gunas*. Information on the potential impact of climate factors on malaria incidence might be helpful to guide malaria prevention programmes aimed at the eventual elimination of this disease, from República de Panamá and Mesoamerica. For example, strategic interventions in the vulnerable region of Guna Yala, should include comprehensive health impact assessments [21, 79, 83] with a focus in areas foreseen to see large influx of climate change triggered *Guna* migration, such as Carti, a site already chosen for island *Guna* population relocation following the disappearance of the Caribbean Islands currently serving as home for most of the *Gunas* living in Guna Yala.

## Additional files

**Additional file 1: Figure S1.** Rainfall time series. (A) Mulatupo (9.000259, − 77.866676) and (B) Nargana (9.444246, − 78.585331). Data are courtesy of ETESA, Panamanian National Electrical Company.

**Additional file 2: Figure S2.** Correlation functions for the 1998–2016 monthly time series. (A) Malaria time series autocorrelation function (ACF) (B) Malaria time series partial autocorrelation function (PACF). Cross correlation function (CCF) between malaria and (C) Niño 4 (D) rainfall (E) temperature. In the plot panels, orange lines indicate the value of the correlation function, the black solid line indicates a correlation value of 0, while the dotted lines indicate 95% confidence intervals within which correlations are expected by chance. Time lags in the x axis of all panels are in months.

**Additional file 3: Figure S3.** Cross correlation function between malaria and SD NDVI. The orange line indicates correlation function values at different time lags (in months), the black solid line indicates a correlation value of 0, while the dotted lines indicate 95% confidence intervals within which correlations are expected by chance.

## Abbreviations

ENSO: El Niño Southern Oscillation
NOAA: National Oceanic and Atmospheric Administration
NDVI: normalized difference vegetation index
LP DAAC: NASA Land Processes Distributed Active Archive Center
EROS: USGS/Earth Resources Observation and Science
ACF: autocorrelation function
PACF: partial autocorrelation function
CCF: cross-correlation function
AIC: Akaike information criterion
AR: autoregressive
SAR: seasonal autoregressive
SD: standard deviation
WHA: World Health Assembly
MDG: Millennium development goals
