# Supplementary figures and images for: Climatic fluctuations and malaria transmission dynamics, prior to elimination, in Guna Yala, República de Panamá
Source: Malar J. 2018 Feb 20;17:85. doi: 10.1186/s12936-018-2235-3 (PMC5819664; doi:10.1186/s12936-018-2235-3)

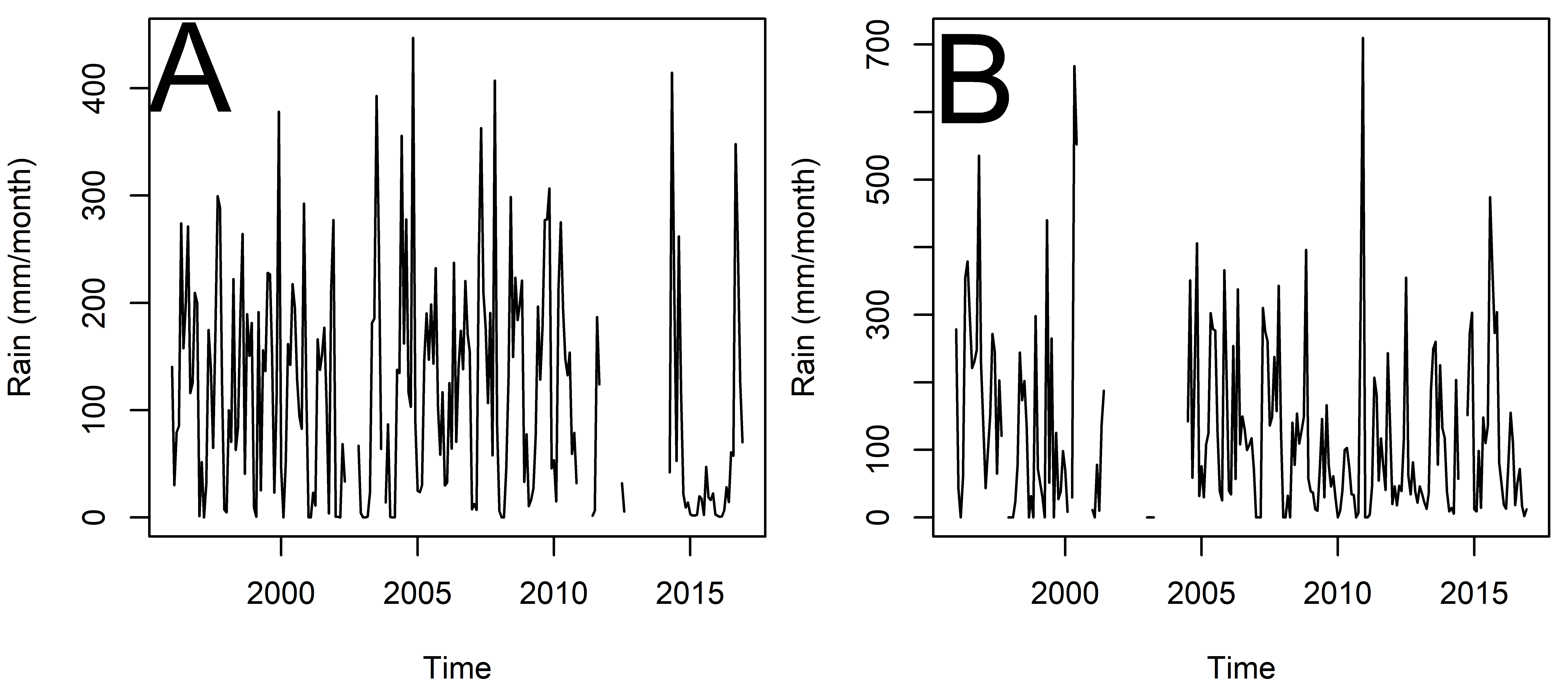

Supplement: Supplementary file 1 — Additional file 1: Figure S1. Rainfall time series. (A) Mulatupo (9.000259, − 77.866676) and (B) Nargana (9.444246, − 78.585331). Data are courtesy of ETESA, Panamanian National Electrical Company. [file 12936_2018_2235_MOESM1_ESM.tiff]

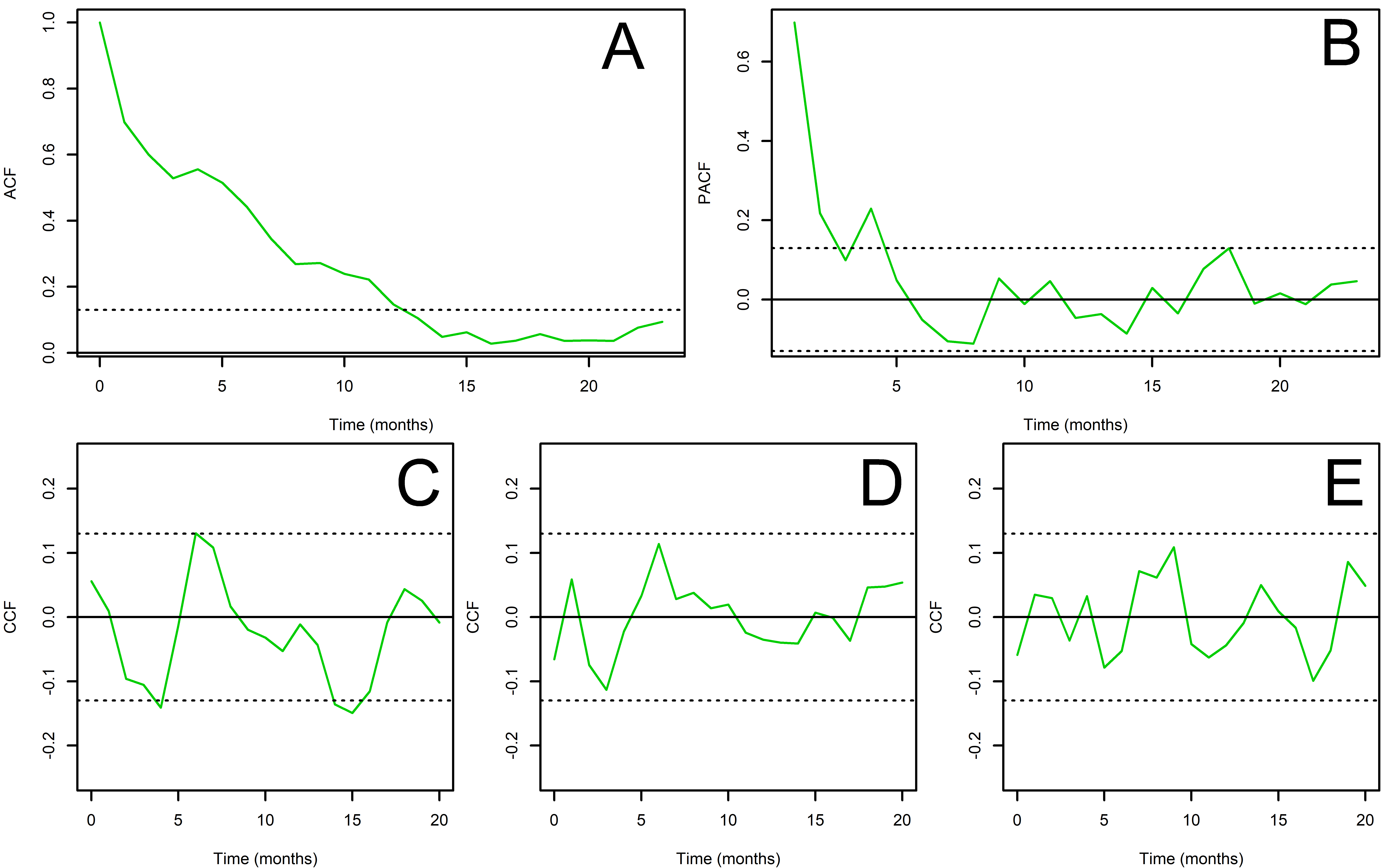

Supplement: Supplementary file 2 — Additional file 2: Figure S2. Correlation functions for the 1998–2016 monthly time series. (A) Malaria time series autocorrelation function (ACF) (B) Malaria time series partial autocorrelation function (PACF). Cross correlation function (CCF) between malaria and (C) Niño 4 (D) rainfall (E) temperature. In the plot panels, orange lines indicate the value of the correlation function, the black solid line indicates a correlation value of 0, while the dotted lines indicate 95% confidence intervals within which correlations are expected by chance. Time lags in the x axis of all panels are in months. [file 12936_2018_2235_MOESM2_ESM.tiff]

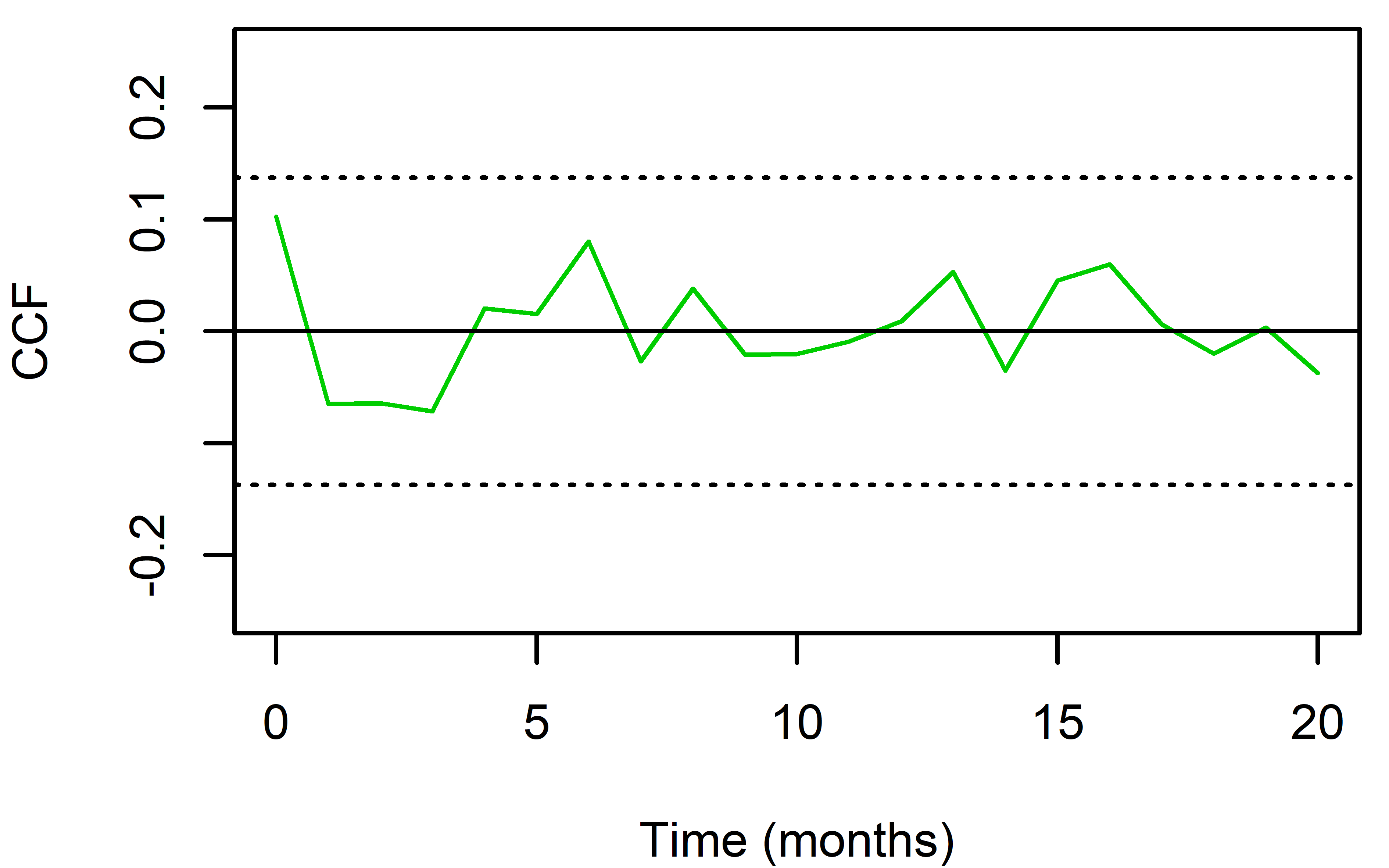

Supplement: Supplementary file 3 — Additional file 3: Figure S3. Cross correlation function between malaria and SD NDVI. The orange line indicates correlation function values at different time lags (in months), the black solid line indicates a correlation value of 0, while the dotted lines indicate 95% confidence intervals within which correlations are expected by chance. [file 12936_2018_2235_MOESM3_ESM.tiff]
